# Nucleotide-dependent assembly of the peroxisomal receptor export complex

**DOI:** 10.1038/srep19838

**Published:** 2016-02-04

**Authors:** Immanuel Grimm, Delia Saffian, Wolfgang Girzalsky, Ralf Erdmann

**Affiliations:** 1 Abteilung für Systembiochemie, Institut für Biochemie und Pathobiochemie, Medizinische Fakultät der Ruhr-Universität Bochum, D-44780 Bochum, Germany.

## Abstract

Pex1p and Pex6p are two AAA-ATPases required for biogenesis of peroxisomes. Both proteins form a hetero-hexameric complex in an ATP-dependent manner, which has a dual localization in the cytosol and at the peroxisomal membrane. At the peroxisomal membrane, the complex is responsible for the release of the import receptor Pex5p at the end of the matrix protein import cycle. In this study, we analyzed the recruitment of the AAA-complex to its anchor protein Pex15p at the peroxisomal membrane. We show that the AAA-complex is properly assembled even under ADP-conditions and is able to bind efficiently to Pex15p *in vivo*. We reconstituted binding of the Pex1/6p-complex to Pex15p *in vitro* and show that Pex6p mediates binding to the cytosolic part of Pex15p via a direct interaction. Analysis of the isolated complex revealed a stoichiometry of Pex1p/Pex6p/Pex15p of 3:3:3, indicating that each Pex6p molecule of the AAA-complex binds Pex15p. Binding of the AAA-complex to Pex15p in particular and to the import machinery in general is stabilized when ATP is bound to the second AAA-domain of Pex6p and its hydrolysis is prevented. The data indicate that receptor release in peroxisomal protein import is associated with a nucleotide-depending Pex1/6p-cycle of Pex15p-binding and release.

Peroxisomes are multifunctional dynamic organelles present in nearly all eukaryotic cells. A set of specific proteins, so called peroxins, are required for biogenesis of these organelles and the maintenance of their function^1^. To date 34 peroxins are known, among them Pex1p and Pex6p, which both represent members of the AAA (ATPases associated with various cellular activities) protein family^2^. In the early 90s, it was recognized that Pex1p, NSF (N-ethylmaleimide-sensitive factor) and p97/VCP (valosin-containing protein) share a highly conserved AAA domain^3^,^4^,^5^. The AAA^+^ family today displays a group of at least 30.000 AAA^+^-proteins found in all biological kingdoms^6^,^7^,^8^,^9^. Pex1p as well as Pex6p belong to the group of classical type II AAA-ATPases, containing two AAA-domains, termed D1 and D2, located downstream to N-terminal domains (NTDs)^10^ (Fig. 1a). The D2 domains of both peroxins display a high conservation with typical features like Walker A- and B-motifs for ATP binding and hydrolysis (A2, B2), beside conserved aromatic pore loops and arginine-fingers in the second region of homology^11^. However, the D1 domains are poorly conserved, exposing a Walker A motif in Pex1p (A1) and a modified Walker A motif in Pex6p (*A1*), together with remnants of Walker B sides (*B1*) most likely not suitable for ATP hydrolysis^11^.

Unlike most other AAA-proteins, which usually form homohexameric structures, Pex1p and Pex6p interact with each other and assemble into a heteromeric-complex like the mitochondrial Yta10p-Yta12p-complex and the six Rpt proteins of the proteasome^12^,^13^. First described in *Pichia pastoris*^14^, the interaction of Pex1p and Pex6p was confirmed in other organisms like *Homo sapiens*^15^, *Hansenula polymorpha*^16^, and *Saccharomyces cerevisiae*^17^. Studies on *S. cerevisiae* Pex1p and Pex6p revealed that ATP-binding to the second AAA-domain of Pex1p is essential for the interaction of both peroxins and the formation of a hetero-hexameric complex^17^,^18^.

It is well established that Pex1p as well as Pex6p shows a dual localization in the cell, as they are located in the cytosol as well as at the peroxisomal membrane. Thereby, organellar association is facilitated by binding of the N-terminal region of Pex6p to the cytosolic domain of the tail-anchored membrane protein Pex15p or its functional orthologues in mammals (Pex26p) and plants (APM9)^19^,^20^,^21^. While at least in yeast this interaction can be enhanced by ATP-binding to the first AAA-domain of Pex6p, its second and conserved AAA-domain seems to be required for the localization of the AAA-complex. It has been shown that ATP-binding as well as ATP-hydrolysis at Pex6p D2 is crucial for the release of the AAA-complex from the peroxisomal membrane as corresponding mutations led to an increased association with peroxisomes^19^. The data indicate that the assembly and disassembly of the AAA-complex as well as its Pex15p-mediated association and release from the membrane are dynamic processes possibly regulated by the nucleotide binding state of the proteins.

In order to gain more insight into this process, we analyzed membrane-attached peroxisomal AAA-complexes at different nucleotide states. Pex1/6p membrane complexes including a Walker B mutation in Pex6p resulted in an enhanced binding to its membrane anchor Pex15p, stronger interactions to different components of the import machinery and accumulation of polyubiquitinated Pex5p. A corresponding Walker B mutation in Pex1p revealed no such effects. We compared these *in vivo* data with experiments using bacterial expressed proteins and reconstituted the binding of the Pex1/6p-complex to Pex15p *in vitro*. Pex6p mediates the binding of the AAA-complex to the cytosolic part of Pex15p via a direct interaction and independent of other proteins. Moreover, our data clarify the direct correlation between ATP hydrolysis in Pex6p and the dissociation of the Pex1/6p-complex from Pex15p.

Based on these observations and considering the bipartite localization of the AAA-complex, we conclude that action of Pex1/6p at the peroxisomal import machinery is linked to an association and releasing cycle between the cytosol and the peroxisomal membrane.

## Results

### Presence of ADP or ATP enables formation of the recombinant AAA-complex

**Figure 1:**
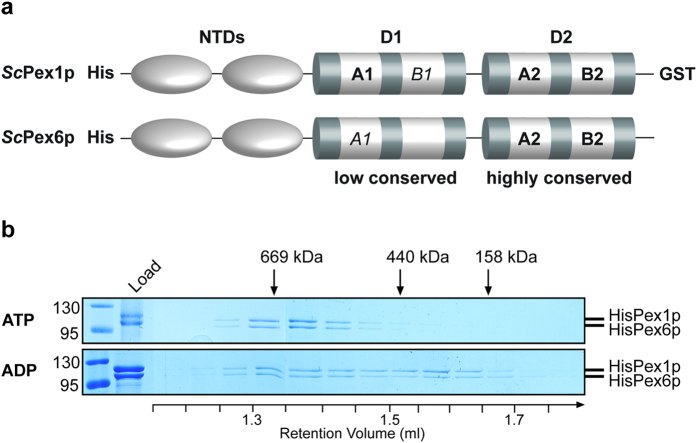
Nucleotide-dependent formation of the Pex1/6p-complex. **(a)** Domain organization of Pex1p and Pex6p. Pex1p and Pex6p are type-II AAA-proteins, which both comprise two N-terminal domains (NTDs), a non-conserved first AAA-domain (D1) and a conserved second AAA-domain (D2). Conserved Walker A motifs (A2) for ATP binding and Walker B motifs (B2) for hydrolysis of ATP are located in the D2 domains of both peroxins. The D1 domains comprise only remnants of Walker A and B motifs (*A1, B1*) with exception of a conserved Walker A motif in Pex1p (A1). **(b)** HisPex1p and HisPex6p in complex were isolated in the presence of ATP or ADP and isolated complexes were analyzed by size exclusion chromatography. Fractions were separated by SDS-PAGE and stained with Coomassie. In the presence of ATP, Pex6p and Pex1p both peaked at a size of approximately 700 kDa which represents the heterohexameric AAA-complex. Under ADP conditions, Pex1p and Pex6p assembled to the 700 kDa complex with a portion disassembling into monomeric Pex6p and trimeric Pex1p.

The AAA-peroxins Pex1p and Pex6p form a heterohexameric complex required in the late step of the peroxisomal matrix protein import. Both peroxins are characterized by the presence of two AAA-domains, termed D1 and D2, downstream to N-terminal domains (NTDs) (Fig. 1a). Two-hybrid studies with the yeast AAA-peroxins indicated that ATP-binding to the D2 AAA-domain of Pex1p is a prerequisite for its interaction with Pex6p^17^. In line with this assumption, we recently demonstrated that the recombinant Pex1/6p-complex is formed only in the presence of ATP, whereas ATP-depletion results in the disassembly of the heterohexameric core complex into smaller subcomplexes^18^. Since the AAA-complex performs its ATPase activity most likely at the peroxisomal membrane to drive the export of PTS-receptors, we addressed the question of how the complex is assembled when ATP is hydrolysed to ADP. To this end, recombinant yeast Pex1p and Pex6p both with an N-terminal hexahistidyl-tag (His6) and Pex1p in addition with a C-terminal glutathione-S-transferase (GST) tag were expressed separately in *E. coli*. The cells were mixed and lyzed and both AAA-peroxins were co-purified by affinity chromatography in presence of either ATP or ADP. First, the His-tagged AAA-proteins were purified together by affinity chromatography with nickel-NTA-agarose (NiNTA). Subsequently, the GST-tagged AAA-complex was subjected to affinity chromatography on GSH-agarose. The bound AAA-complex was released by thrombin cleavage with the GST-tag remaining on the column, resulting in Pex1p/Pex6p complexes with high purity. For estimation of the rate of complex-formation, the recombinant AAA-complex was further subjected to size exclusion chromatography on Superose6 column. Analysis of the eluted proteins by SDS-PAGE revealed that in presence of ATP the AAA-proteins formed a complex with a size of approximately 700 kDa (Fig. 1b), which is in line with previous findings^10^,^11^,^18^,^22^. Although a portion of the complex was not completely assembled in the presence of 5 mM ADP, the major part of Pex1p and Pex6p co-migrated with a peak at 700 kDa, indicating that the proteins assembled to the heterohexameric complex also in the presence of ADP (Fig. 1b). From this we conclude that after hydrolysis of ATP to ADP, the AAA-peroxins can still associate to form an oligomeric complex, which might arrange in a looser compilation with a tendency to dissociate.

### Nucleotide dependent *in vivo* binding of the AAA-complex to Pex15p

**Figure 2:**
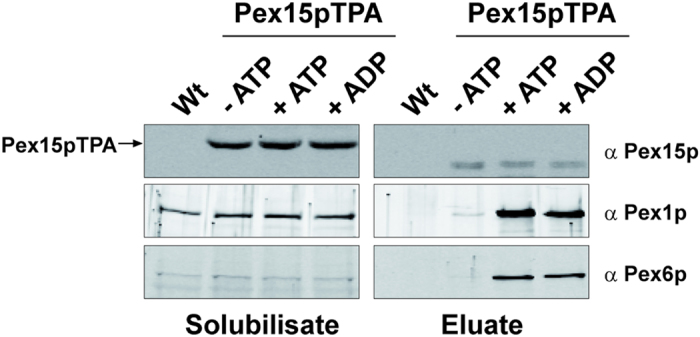
Nucleotide-dependent association of the AAA-complex to Pex15p. Protein complexes were isolated from 1% digitonin-solubilized membranes of wild-type (control) and wild-type cells expressing Pex15pTPA via IgG-Sepharose and subsequent TEV protease cleavage. Complex isolation was performed in the absence or presence of 5 mM ATP and ADP as indicated. Equal portion of solubilisate (1.2% of total) and eluate (20% of total) were subjected to SDS-PAGE and immunoblot analysis and probed with specific antibodies as indicated. Pex1p and Pex6p were part of the Pex15p complex when ADP or ATP was present, whereas a significant loss of binding to Pex15p was observed in the absence of nucleotides.

The Pex1/6p-complex is localized in the cytosol and it is found associated with the peroxisomal membrane. The recruitment to yeast peroxisomes is mediated by the tail-anchored peroxisomal membrane protein Pex15p via binding of the N-terminal domain of Pex6p^19^. We addressed the question whether the nucleotide state affects the Pex15p binding *in vivo* as it was observed *in vitro* for the AAA-complex assembly. To this end, a wild-type strain expressing Pex15p genomically tagged with protein A (Pex15pTPA), which harbors a *tobacco etch* virus (TEV) cleavage site between both fusion-partners was used^23^. The Pex15pTPA complex was isolated after 1% digitonin extraction from total membranes by IgG-Sepharose affinity chromatography in the absence or presence of 5 mM ATP and ADP. Obtained TEV protease eluates were subjected to SDS-PAGE and analyzed by immunoblotting. None of the analyzed proteins was isolated from the untransformed wild-type strain, which served as control for the specificity of the isolation procedure (Fig. 2). Pex15p was isolated via its TPA-tag to the same extent independent of the nucleotide state of the samples. Pex1p as well as Pex6p were co-purified with Pex15p in presence of ATP and slightly reduced (to approximately 70% of ATP-conditions as judged by signal intensity measurements) in presence of ADP. In clear contrast, the amount of AAA-peroxins in the eluate was drastically reduced in the absence of ATP or ADP (Fig. 2). This demonstrates that the presence of either ATP or ADP is required for efficient binding of the AAA-complex to Pex15p. Moreover, it seems that less efficient interaction takes place in the presence of ADP compared to ATP.

### Impact of Walker B mutations on Pex1/6p membrane complexes

Since the Pex1/6p complex consistently hydrolyses ATP to ADP and phosphate, we do not know the exact nucleotide state of the isolated complex in presence of ATP. To fix the complex in the ATP-bound state, we analyzed Walker B mutants of the membrane attached Pex1/6p complex. Recently, we showed that Pex1p and Pex6p contribute to the overall ATP hydrolysis rate of the AAA-complex in an unsymmetrical manner. While a mutation of the conserved glutamate in the Walker B motif of Pex1p (PEX1WB^E798Q^) resulted in a slight decrease of the ATPase activity, a corresponding mutation in Pex6p (PEX6WB^E832Q^) abolished hydrolysis activity completely^11^. In line with this observation, yeast strains harboring the Pex6p Walker B mutation PEX6WB^E832Q^ do not grow on media with oleate as sole carbon source, whereas Pex1p Walker B mutants PEX1WB^E798Q^ are able to utilize oleic acid^11^. Interestingly, a mutation of the adjacent conserved aspartate in the Pex1p Walker B motif PEX1WB^D797Q^ 17 or PEX1WB^D797N^ show a clear growth defect on oleic acid medium (Supplementary Fig. 1a). These data indicate that the glutamate E798 and thus ATP-hydrolysis at the conserved AAA-domain is not essential for Pex1 function, while mutation of the adjacent aspartate D797 is essential for the function of the protein. To investigate the reason for the different functional activity of the Pex1p mutants, we purified recombinant Pex1/6p, Pex1WB^D797N^/6p and Pex1WB^E798Q^/6p complexes by tandem affinity chromatography and size exclusion chromatography and tested the stability of the heterohexameric complexes after incubation at 37 °C by a second gel filtration. Whereas, wild-type Pex1/6p and Pex1WB^E798Q/^6p complexes remained in a hexameric conformation, Pex1WB^D797N^/6p rapidly dissociates into typical pattern of trimeric Pex1p and monomeric Pex6p (Supplementary Fig. 1b). From this we conclude that mutation of the aspartate D797N has a negative effect not only on ATP hydrolysis, but also on the AAA complex stability, probably by interfering with a proper nucleotide binding in Pex1p D2. Thus, the observed phenotype of Pex1pWB^D797N^ cells on oleic acid is not attributed to a defect of ATP hydrolysis in Pex1p, but to a defect in AAA complex formation. Therefore, we decided to use the Pex1pWB^E798Q^ variant for further experiments. Exchange of this conserved glutamate to glutamine was shown for Pex6p^11^,^22^, and for many other AAA-proteins to inhibit ATP hydrolysis but not ATP binding^8^.

**Figure 3:**
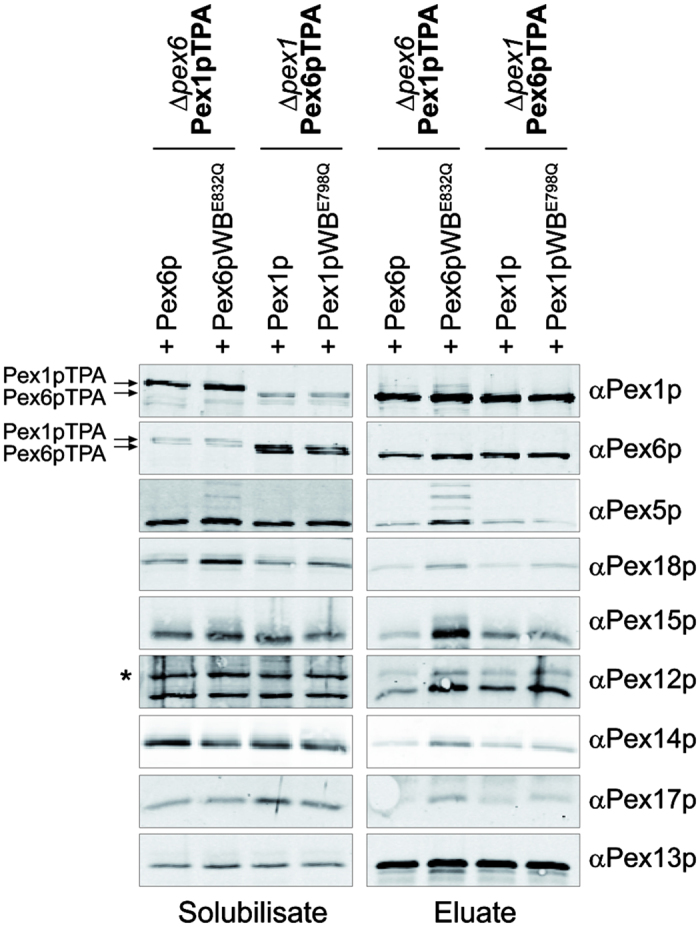
Impact of Walker B mutations in Pex1p or Pex6p on membrane complex assembly. Membrane associated complexes from Δ*pex1* or Δ*pex6* cells complemented with Pex1p, Pexp1WB, Pex6p, or Pexp6WB, respectively were analyzed. Protein complexes were isolated from 1% digitonin-solubilized membranes using Pex1pTPA or Pex6TPA fusion proteins via IgG-Sepharose and subsequent TEV protease cleavage in presence of 5 mM ATP. Equal portion of solubilisate (0.2% of total) and eluate (15% of total) were subjected to SDS-PAGE and immunoblot analysis and probed with specific antibodies as indicated. Expression of Pex6pWB leads to an accumulation of Pex5p with typical polyubiquitination pattern, a strong increase of Pex15p as well as slight increased signals for Pex18p (PTS2 co-receptor), Pex12p (part of RING-finger complex) and Pex14p/Pex17p (part of the docking machinery). In contrast, Pex1pWB-cells display no obvious differences in comparison to the wild type situation (+Pex6p, +Pex1p). Arrows indicate Protein A fusion proteins which are both recognized by αPex1p and αPex6p antibodies. *Double-bands of Pex12p are caused by strong intramolecular disulfide bonds between the seven RING finger-like cysteines^46^.

To investigate the effect of ATP hydrolysis of Pex1p and Pex6p on the formation and stability of AAA membrane complexes, we complemented genomically modified Δ*pex1*Pex6pTPA and Δ*pex6*Pex1pTPA strains with plasmids coding for PEX1 and PEX1WB^E798Q^ or PEX6 and PEX6WB^E832Q^, respectively, under control of their own promoters. AAA-complexes were solubilized with 1% digitonin from total membrane pellets, isolated by IgG-Sepharose affinity chromatography in presence of 5 mM ATP and analyzed by SDS-PAGE and immunoblotting (Fig. 3). In all cases, eluates showed similar amounts of isolated Pex1p and Pex6p. The AAA-complexes were associated with its membrane anchor Pex15p and different members of the peroxisomal matrix protein import machinery, namely the PTS receptors Pex5p and Pex18p, the RING-finger complex represented by Pex12p and members of the importomer (Pex14p, Pex17p, Pex13p). In comparison to the wild-type situation (Δ*pex6*Pex1pTPA +Pex6p, Δ*pex1*Pex6pTPA +Pex1p), the Walker B mutation in Pex1p (Δ*pex1*Pex6pTPA +Pex1pWB^E798Q^) did not show any detectable effect on membrane complex formation. In contrast, when Pex6p was mutated (Δ*pex6*Pex1pTPA +Pex6pWB^E832Q^), a clear polyubiquitinylation pattern of accumulated Pex5p was discernible. This reflects the oleate growth defect and loss of ATPase activity of the Pex1/6p Walker B mutants^11^. Likewise, the increased amount of Pex18p in the solubilisate and complex eluate indicates that the WB mutation of Pex6p results in an import defect, which for Pex18p is known to result in its membrane association and decreased degradation^24^,^25^. Besides Pex5p and Pex18p, nearly all components of the import machinery exhibited an increased presence in the Pex1/6pWB complex when compared to wild type level. In particular, the abundance of the AAA-membrane anchor Pex15p was profoundly increased. Taken together, these results indicate that the AAA-complex strongly associates to Pex15p when Pex6p is fixed in a constantly ATP bound state. Moreover, the loss of Pex1/6p ATPase activity blocks the export of the PTS receptors and stabilizes the interaction between the receptor import- and export-machinery.

### Association of Pex15p with the recombinant AAA-complex

**Figure 4:**
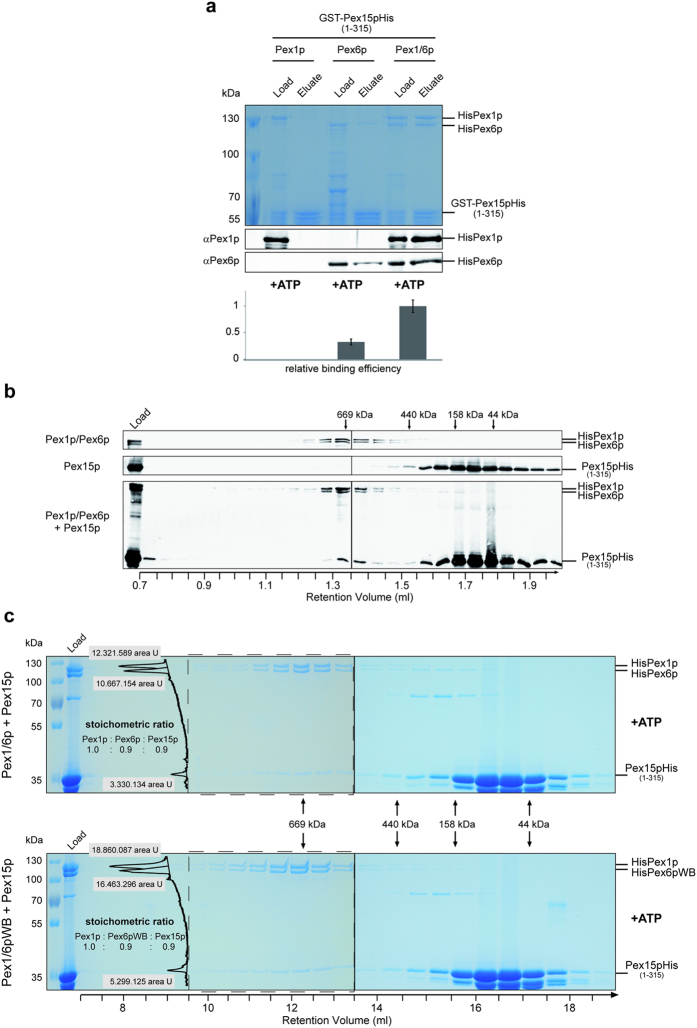
*In vitro* assembly of the recombinant Pex1p/Pex6p/Pex15p-complex. **(a)** The cytoplasmic domain of purified recombinant Pex15p (GST-Pex15p1-315His) was combined with HisPex1p, HisPex6p, or with the hexameric Pex1/6p complex in presence of 5 mM ATP and subjected to GSH pull down assay. Load and 5x concentrated eluate fractions were separated by SDS-PAGE and proteins were visualized by Coomassie staining (upper panel) or with specific antisera as indicated (middle panel). Pex6p signals from load and eluate fractions were measured and relative binding efficiency determined (lower panel) by setting Pex1/6p binding-capacity to 1. Error bars represent standard error of mean (SEM) of four independent experiments. In complex with Pex1p, Pex6p binds three times stronger to Pex15p than single Pex6p. **(b)** Pex1/6p, Pex15p(1-315) or the Pex1/6p/Pex15p-complex of the three proteins were subjected to size exclusion chromatography in presence of ATP. Collected fractions were subjected to SDS-PAGE and immunoblot analysis and indicated proteins were detected via their His-tag with anti-His-antibodies. Pex15p was shifted to fractions of about 700 kDa in the presence of the AAA-complex, demonstrating its association with this complex. **(c)** Size exclusion chromatography of the Pex1/6p/Pex15p-complex (upper panel) and the Pex6p Walker B mutated version Pex1/6pWB/Pex15p (lower panel) in presence of ATP after isolation of the proteins by GSH pull down assay as described in (a) and subsequent thrombin elution. Fractions were analyzed by SDS-PAGE and Coomassie staining and signal intensities of peak fraktions (dashed boxes) were evaluated using ImageJ. Taking into account the molecular weights of Pex1p (117 kDa), Pex6p (115 kDa) and Pex15p1-315 (36 kDa), both complexes consist of equal Pex1p, Pex6p and Pex15p ratios.

To gain more insight into the recruitment of the AAA-complex to and liberation from the peroxisomal membrane, we investigated the interaction of the recombinant Pex1/6p-complex with the cytosolic domain of Pex15p, comprising amino-acid residues 1 to 315 (Pex15p_1-315_). To this end, GST-Pex15p_1-315_-His was expressed in *E. coli* and purified by GSH-agarose affinity chromatography. Glutathione, which was used to elute Pex15p_1-315_ from the affinity matrix, was removed by dialysis. Subsequently, equal portions of the obtained Pex15p_1-315_ were incubated with purified Pex1p and Pex6p or Pex1p/Pex6p-complex. The assays were again loaded on GSH-agarose columns and Pex15p-complexes were released by glutathione. Load and eluted fractions were subjected to SDS-PAGE and proteins were stained with Coomassie, the presence of Pex1p and Pex6p was analyzed by immunoblotting (Fig. 4a). The data show that a significant portion of Pex6p is retained on the column in the presence of GST-Pex15p, whereas Pex1p alone is not, indicating that isolated Pex6p but not Pex1p is capable to bind Pex15p directly. In the same experimental setup, the Pex1/6p complex is retained on the column, indicating that the AAA-complex is efficiently bound by Pex15p (Fig. 4a). The soluble AAA-complex is a heterohexamer formed by a trimer of Pex1/6p dimers^10^,^11^,^18^,^22^. Equal portions of Pex1p and Pex6p bound to Pex15p, indicating that the Pex1/6p complex remains unchanged in composition, when recruited from the cytosol to the peroxisomal membrane and that no additional factors are required for its binding to Pex15p. Signal intensity measurements of Pex6p present in the load and in eluate fractions revealed striking increase when the Pex1/6p-complex was loaded in comparison to the single protein. This result indicates that the interaction of Pex15p and Pex6p is increased when Pex6p is in complex with Pex1p (Fig. 4a, lower panel).

To find out how many Pex15p molecules are associated with the Pex1/6p AAA-complex, we analyzed the composition of the purified complex of Pex15p and the AAA-peroxins in detail. The complex was isolated by affinity chromatography and by size exclusion chromatography. To exclude dimerization effects of GST-tagged Pex15p, the complex was released from the GSH-agarose by thrombin cleavage with the GST-tag remaining on the column. Gel filtration revealed that the complex of Pex1p, Pex6p and Pex15p has a size of about 700 kDa (Fig. 4b), whereas Pex15p alone was detected in fractions of about 100 kDa. A shift of the Pex1/6p-complex in size upon binding to Pex15p was not visible, due to the low resolution of the gel filtration column in this size area. However, when the eluted proteins were analyzed by immunoblotting with anti-His antibodies, which detects the remaining His-tag of all three proteins, HisPex1p, HisPex6p and Pex15pHis, it is evident that a portion of Pex15p shifted to the fractions of the AAA-complex. Similar intensities of the signals of the 700 kDa-complex indicate that the complex contains equal amounts of Pex1p, Pex6p and Pex15p. These data suggest that each Pex6p molecule is capable to bind one Pex15p, resulting in a 3:3:3 ratio of the Pex1p/Pex6p/Pex15p complex.

In order to verify the stoichiometry of the Pex1p/Pex6p/Pex15p complex, we performed the isolation procedure in higher quantity and analyzed the obtained complex on a preparative Superose6 column. Fractions of the size exclusion chromatography were analyzed by SDS-PAGE and Coomassie staining. As observed before, the AAA-complexes peaked in fractions of a size of about 700 kDa and contained a significant portion of co-migrating Pex15p_1-315_His (Fig. 4c, upper panel). In parallel, a complex with Walker B mutation in Pex6p was analyzed (Pex1/6pWB +Pex15p) (Fig. 4c, lower panel). Both wild-type and Pex1/6pWB/Pex15p complexes showed same elution profiles, although the amount of the mutated complex was substantially increased. Intensity measurements of Pex1p- Pex6p- and Pex15p- signals revealed a stoichiometric ratio of 1:0.9:0.9 for Pex1p, Pex6p and Pex15p in both formed complexes. Indeed, this data indicate that each Pex6p molecule is capable to bind one Pex15p, resulting in a 3:3:3 ratio of the Pex1p/Pex6p/Pex15p complex.

### ATP-hydrolysis triggers the dissociation of Pex15p and the AAA-complex

**Figure 5:**
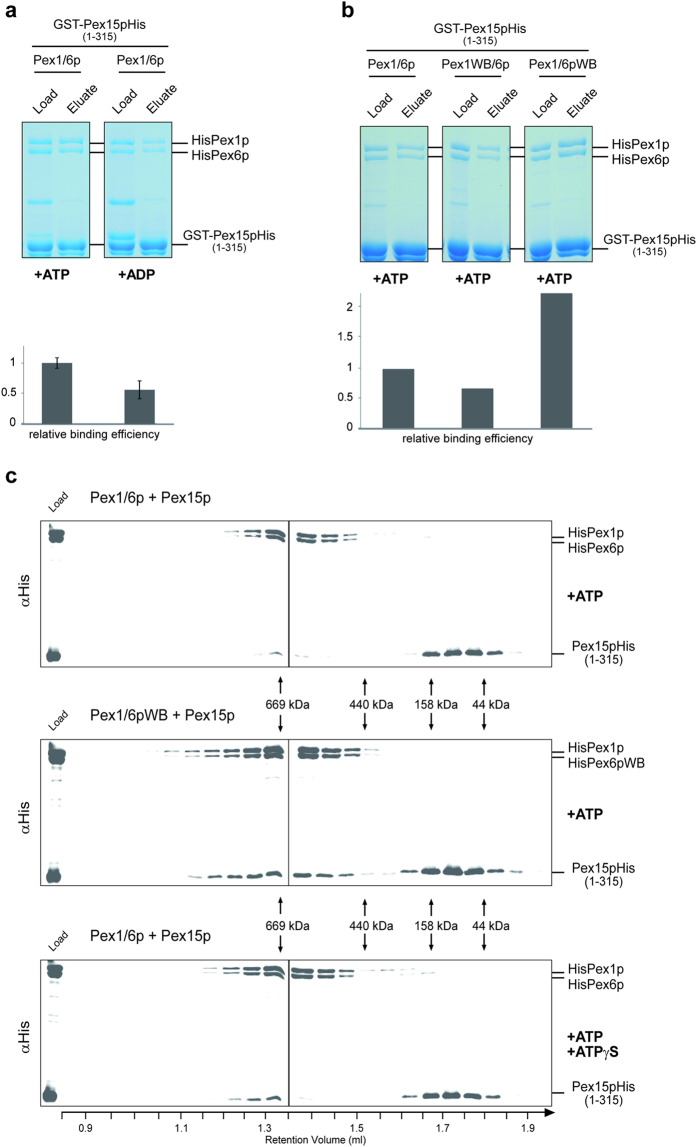
Nucleotide-dependent association and dissociation of the reconstructed Pex1p/Pex6p/Pex15p-complex. **(a)** Hexameric Pex1/6p complexes were isolated in presence of ATP or ADP and incubated with GST-Pex15p1-315His and then subjected to GST pull down assay. Load and 5x concentrates of eluate fractions were analyzed by SDS-PAGE and Coomassie staining (upper panel) and relative binding efficiency (lower panel) was estimated by measuring Pex6p signals from load and eluate fractions and setting Pex1/6p +ATP binding-capacity to 1. **(b)** GST-Pex15p1-315His pull down assay in presence of ATP using hexameric Pex1/6p wild type, Pex1WB/6p and Pex1/6pWB complexes. Binding efficiency was determined as in (**a**). In contrast to wild-type complex, Walker B-mutation of Pex6p leads to a significantly higher association of the complex with Pex15p, indicating that ATP-hydrolysis triggers complex-dissociation. **(c)** 700 kDa Pex1p/Pex6p/Pex15p and Pex1p/Pex6pWB/Pex15p complexes as well as Pex1p/Pex6p/Pex15p wild-type complexes, which had been incubated with 2.5 mM ATPγS during the isolation procedure, were obtained from size exclusion chromatography. Afterwards these complexes were incubated 72 h at 4 °C and subjected again to size exclusion chromatography. Presence of proteins was monitored by SDS-PAGE and immuno-blotting using anti-his-antibodies. Pex1p/Pex6pWB as well as wild-type complexes incubated with ATPγS exhibit a stabilized interaction to Pex15p, proving the direct correlation between ATP hydrolysis and complex-dissociation.

We next studied the role of nucleotides in the binding of the recombinant Pex1/6p-complex to Pex15p. To this end, we analyzed the binding of isolated GST-Pex15p_1-315_His to wild-type Pex1/6p-complexes in presence of ATP and ADP, and analyzed whether mutations of Walker B motifs of the AAA-proteins have an influence on binding of the complex to Pex15p. Complexes were bound to GSH-agarose, eluted from the columns by addition of reduced glutathione and visualized by SDS-PAGE and Coomassie staining (Fig. 5a,b, upper panels). Signal intensity measurements of the AAA-proteins present in the load and the eluate fraction were performed and the ratio obtained for wild-type complex under ATP-condition normalized to a relative binding-capacity of one (Fig. 5a,b, lower panels). When ADP was present instead of ATP, the binding of the wild-type AAA-complex to the recombinant Pex15p was reduced (Fig. 5a), which corroborates the observed binding behavior of the isolated endogenous Pex15p-complex (Fig. 2). A reduced binding was also observed for the complex composed of wild-type Pex6p and mutated Pex1p (Pex1WB/6p, Fig. 5b). However, when ATPase activity of the AAA-complex was blocked by mutation of Pex6p (Pex1/6pWB), the binding capacity of the formed AAA-complex was significantly increased (Fig. 5b). This is in agreement with the observed accumulation of Pex15p at Pex1/6pWB-membrane complexes isolated from yeast (Fig. 3). We conclude that hydrolysis of ATP to ADP via the D2-domain of Pex6p weakens the interaction to Pex15p and thus might be a regulatory step in the AAA-cycle.

Driven by our observation that the *in vivo* and *in vitro* association of the AAA-complex with Pex15p is strengthened when ATP-hydrolysis in the Pex6p D2-domain is prevented, we analyzed the stability of the complexes over a longer period of time. Isolated wild-type Pex1/6p as well as the mutated Pex1/6pWB were bound to GSH-agarose loaded with GST-Pex15p_1-315_His. The Pex15p_1-315_His and bound AAA-peroxins were eluted by thrombin cleavage. In a third approach 2.5 mM ATPγS was added during thrombin incubation of Pex1/6p wild-type proteins bound to Pex15p_1-315_His. The eluted complexes were further purified by size exclusion chromatography. Subsequently, the 700 kDa fractions were pooled and kept on ice for 72h, before they were subjected again to size exclusion chromatography. After this time-period, wild-type Pex1p and Pex6p were detected unaltered at 700 kDa fractions, whereas most of Pex15p_1-315_His was found in later fractions with a size of about 100 kDa (Fig. 5c, upper panel). Obviously, after incubation on ice, the AAA-complex remains assembled but dissociates from Pex15p. The complex composed of wild-type Pex1p and the Walker B mutant variant Pex6pWB behaved differently. Here a significant portion of Pex15p_1-315_His was still associated with the AAA-complex (Fig. 5c, middle panel). The Pex1/6/15p complex, which was incubated with a portion of ATPγS displayed an intermediate state (lower panel). Signal intensity measurements of the ratio of associated and dissociated Pex15p molecules revealed that under wild-type conditions less than 10% of Pex15p was still bound to the complex. Block of ATP hydrolysis using the Pex6WB mutant or application of ATPγS did result in a stabilization of the Pex15p association. Under these conditions, the amount of associated Pex15p increased to 40% or 20%, respectively. We conclude that a block of ATP-hydrolysis in the D2 AAA-domain of Pex6p prevents the release of the AAA-complex from its membrane anchor Pex15p.

## Discussion

Pex1p and Pex6p belong to the AAA-protein family^2^,^7^ and are essential components of the peroxisomal protein import machinery^26^,^27^. Despite a similar architecture, they do not perform overlapping functions and are both indispensable for peroxisomal matrix-protein import. They were shown to function as dislocases facilitating the export of the PTS1-receptor Pex5p from the peroxisomal membrane back to the cytosol at the end of an import cycle^28^,^29^. In contrast to the majority of AAA-ATPases, Pex1p and Pex6p assemble into a heteromeric complex^23^. This complex exhibits a dual localization in the cell with a portion attached to the peroxisomal membrane via the membrane protein Pex15p (Pex26p in humans, APM9 in plants)^19^,^20^,^21^, and a fraction localized to the cytosol.

Recently, we and others analyzed the structure of the heterohexameric Pex1/6p complex by electron microscopy^10^,^11^,^22^ and demonstrated that it is assembled as a trimer of Pex1p/Pex6p dimers, resulting in a tight organization with alternating Pex1p and Pex6p subunits. The D1- as well as the D2- domains of Pex1p and Pex6p are organized into two rings arranged on top of each other with a dynamic central pore. The presence of essential aromatic pore loops, which move up and down during the Pex1/6p ATP hydrolysis cycle led to the proposal that Pex5p is actively pulled out of the membrane by a threading mechanism through this central channel, similar to the mechanism of Clp-ATPases^11^,^30^,^31^,^32^. Beside movement of the pore loops, it is likely that the ATPase-cycle also regulates the assembly of the AAA-complex as well as its association with the peroxisomal membrane. The first assumption is supported by the observations that ATP-binding is essential for Pex1/6p-association^17^,^33^, whereas nucleotide depletion results in complex disassembly^18^,^34^. Here we analyzed the influence of the nucleotide bound-state on assembly of the AAA-complex in the cytosol and on its interaction with Pex15p, which targets the complex to the peroxisomal membrane. We demonstrate that the heterohexameric complex arrangement is largely maintained even under excessive ADP conditions (Fig. 1b). This is in contrast to the nucleotide-free or AMP-bound states of the complex, which disassembles in short time^18^. The stabilization of the complex by ADP indicates that the composition of the complex is not altered during the nucleotide cycle but that the complex remains assembled when ATP is hydrolyzed by the intrinsic ATPase activity of the Pex1/6p-complex. Nevertheless, in comparison to the ATP-bound form, the ADP bound AAA-complex showed a tendency to disassemble (Fig. 1b), which is in agreement with the considerably looser conformation as depicted by electron microscopy^11^ and keeps open a possibility for a regulative Pex1/6p disassembly mechanism. While in mammalian cells, the AAA-complex seems to disassemble in the cytosol to form a homotrimeric version of Pex1p^33^, the yeast complex remains assembled in the cytosol^23^. ATP hydrolysis in the D1 domain of human Pex1p is discussed to trigger dissociation of the Pex1/6p complex^34^. However, the D1 domain in yeast Pex1p does not comprise a functional Walker B motif, which might explain that the cytosolic yeast complex remains assembled.

Here we studied the regulation of the recruitment of Pex1/6p to its membrane anchor Pex15p and analyzed the membrane association of AAA-complexes at different nucleotide states *in vivo* and the binding of the recombinant Pex1/6p-complex to Pex15p *in vitro*. Block of ATP-hydrolysis and therefore fixation of Pex6p in the ATP-bound form increased membrane association of the AAA-complex *in vivo* and binding to Pex15p *in vitro* (Figs 3 and 5b). In contrast to the Pex6p Walker B mutation, a corresponding mutation in Pex1p showed no impact on complex formation and composition (Figs 3 and 5b). This distinction reflects the different contributions of Pex1p and Pex6p to the overall ATPase activity of the AAA-complex, which is predominantly attributed to Pex6p^11^. This seems to be a species-independent feature as the import of GFP-PTS1 into human peroxisomes is not affected by a Walker B mutation in Pex1p D2, while it was abolished in Pex6p Walker B cell lines^33^. However, import of catalase was shown to be impaired for both Pex1/6p Walker B variants, indicating that ATP hydrolysis in Pex1p is somehow required at least for the transport of this human protein^33^.

Reconstitution of the Pex1/6/15p complex revealed that Pex6p mediates the binding of the AAA-complex to Pex15p via a direct interaction and independent of other proteins (Fig. 4a), which is in line with previous reports^19^,^23^. Binding assays and size exclusion chromatography indicated that stoichiometric amounts of Pex1p/Pex6p are bound to Pex15p with a ratio of 3:3:3 (Fig. 4b,c). Thus, each of the three Pex6p molecules within the Pex1/6p-complex is capable to anchor the AAA-complex to the peroxisomal membrane by Pex15p-binding. As the affinity of the Pex1/Pex6-complex to Pex15p is lower under ADP condition, we cannot exclude a change of the stoichiometry upon ATP hydrolysis. However, for p97, a similar binding ratio of three p47 adaptor molecules to the homohexameric AAA-complex was found^35^ and also binding of three α-SNAP molecules to three N-terminal domains of the NSF hexamer is discussed^36^,^37^.

Our data suggest that ATP hydrolysis in Pex6p and the dissociation of the Pex1/6p complex from Pex15p is directly correlated. Obviously, the intrinsic ATPase activity of the complex hydrolyses ATP and converts the complex into the ADP-bound form, which exhibits lower Pex15p-binding affinity. Interestingly, also the Pex1WB/6p complex showed lower affinity to Pex15p compared to the wild-type complex, which might indicate that Pex6p preferably is situated in a post-hydrolysis conformation when Pex1pWB is constantly bound to ATP (Fig. 4c). Similar observations where obtained when conformational changes of Pex1/6p complexes were analysed by electron microscopy. Here a Walker B mutation in Pex1p seemed to urge the adjacent Pex6p subunit in a post-hydrolysis conformation^11^. Different to Pex1WB/6p, the inactive Pex6pWB mutant stabilized the complex (Fig. 5b,c) and the amount of associated Pex15p was significantly increased upon block of hydrolysis of Pex6p but not of Pex1p (Fig. 3). In contrast, it was shown that upon block of ATP hydrolysis of human Pex6p, the association of the AAA-complex to the membrane-bound Pex26p, the human counterpart of Pex15p, remained virtually unchanged^34^. Interestingly, peroxisomal targeting of human Pex1p required ATP hydrolysis at the corresponding D1-domain, which led to the conclusion that Pex1p and Pex6p translocate to human peroxisomes in a mutually distinct manner^34^. However, yeast Pex1p does not contain a functional Walker B motif in its D1 domain, and block of ATP-hydrolysis in Pex1p D2 did neither alter the composition of the isolated complexes of the AAA-proteins and Pex15p *in vivo* neither did it affect formation of the complex *in vitro* (Figs 3 and 5b). Our data suggest that the heterohexameric Pex1p/Pex6p-complex in yeast forms in the cytosol and targets to the peroxisomal membrane in a Pex15p- and ATP-dependent manner.

Recently, it was shown that Pex15p can down-regulate the ATPase activity of the AAA-complex, probably via an intermolecular connection of Pex15p to the N-terminal domains of Pex6p, which further contacts the D2 domain of Pex1p and thereby inhibits Pex1p activity^22^. Besides Pex15p, other interaction partners might influence and regulate the ATPase activity of the AAA-complex and might intervene in the dissociation mechanism. Our data are clear in that ATP-hydrolysis weakens the interaction of the Pex1p/Pex6p complex and Pex15p *in vitro* and *in vivo*. However, we cannot exclude that the release of the AAA-complex from Pex15p and from the membrane will require additional factors. Possible candidates are known interaction partners of Pex1/6p like its substrate, the PTS1-receptor Pex5p, and its de-ubiquitinating enzyme Ubp15p in yeast or the export stimulation factor AWP1 in human cells^38^,^39^. In particular, contact of substrate to the AAA-machinery might trigger its activity, similar to other AAA-ATPases^40^. The intrinsic ATPase activity of NSF for instance is very low^41^. Binding of NSF to α-SNAP stimulates its ATPase activity and maximum stimulation is achieved when both α-SNAP and SNARE complexes are included^37^.

**Figure 6:**
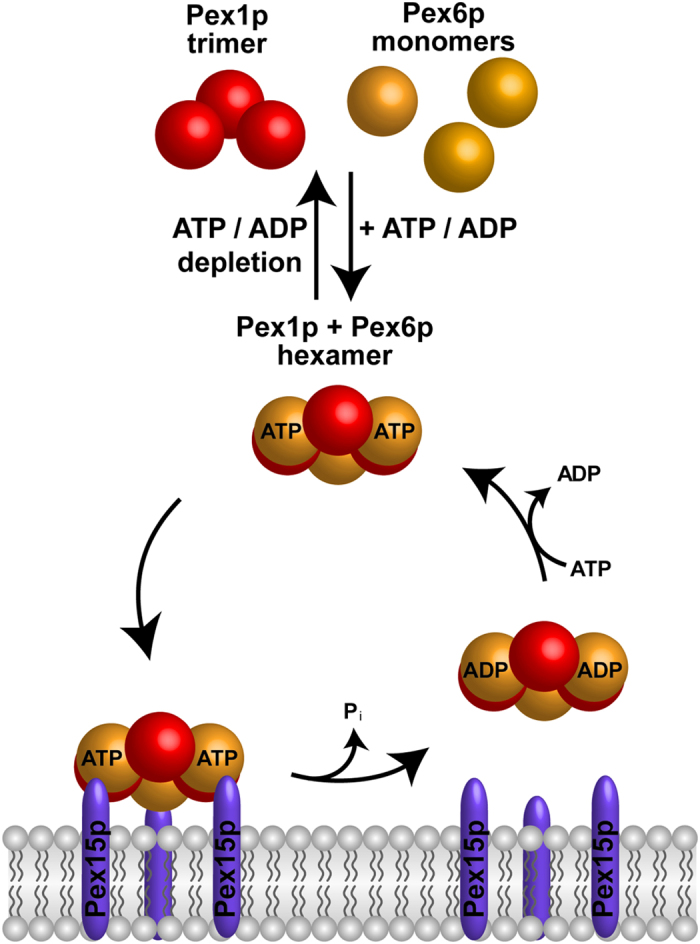
Model for formation and cycle of the Pex1p/Pex6p/Pex15p-complex. The cytosolic heteromeric complex composed of one Pex1p-trimer and three Pex6p-monomers assembles in the presence of ATP in the cytosol. This hexameric complex is recruited to the peroxisomal membrane via binding of Pex6p to the peroxisomal tail-anchor protein Pex15p. This binding is mediated by the N-terminal domains of Pex6p, which directly interact with the cytosolic domain of Pex15p. Hydrolysis of bound ATP in Pex6p results in a detachment of the AAA-complex from Pex15p most likely coupled with the export of PTS-receptors of the peroxisomal matrix protein import machinery.

The current data suggest the following scenario for the Pex1/6p-cycle in yeast (Fig. 6). In the cytosol, newly synthesized Pex1p assembles into a trimer, whereas Pex6p remains monomeric. Binding of ATP to the subcomplexes promotes the assembly of the heterohexameric Pex1p/Pex6p-complex, which represents the dominant state in the cell. The ATP-loaded complex attaches to the peroxisomal membrane via its membrane-anchor protein Pex15p. Contact to Pex15p is mediated by the N-terminal domains of Pex6p, which are located at the three vertices of a triangular shaped structure. At the membrane, the complex functions as dislocase for the PTS1-receptor Pex5p and the energy for the export of Pex5p is supposed to be provided by hydrolysis of ATP. In this context, it is interesting to note that human Pex26p binds the pore constituents Pex14p and Pex5p and that Pex14p can be released from Pex26p in a Pex1p/Pex6p-dependent manner^42^. The ADP-bound form of Pex6p has a reduced binding to Pex15p, which weakens the Pex15p-association of the complex. However, not all Pex6p-subunits of the complex might hydrolyze ATP at the same time, leaving room for a sequential binding and releasing of the three Pex6p molecules, which might be combined with a gradual removal of Pex5p from the membrane. A combination of rotating and pulling movements is discussed for NSF, which together with its adaptor α-SNAP untwists and segregates the SNARE complex^37^,^43^,^44^. At the peroxisomal membrane, such a rotational process might trigger the dissociation of Pex5p from other pore constituents. Finally, the complex is released to the cytosol and exchange of ADP by ATP enables a new round of the AAA-complex cycle.

## Material and Methods

### Plasmids

For expression of yeast HisPex1p, HisPex1pGST and HisPex6p in *Escherichia coli*, plasmids pRSFDuet-HisPex1, pRSFDuet-HisPex1cGST and pRSFDuet-HisPex6 were used^18^. For yeast expression, PEX1 and PEX6 ORFs together with their 5′ and 3′ flanking regions were placed into pRS416 vector according to^17^ (pRS416-Pex1p, pIB1/1) and^19^ (pRS416-Pex6p, pBM34). Constructs for the expression of Pex1p Walker B mutants were obtained by site-directed mutagenesis using pRSFDuet-HisPex1cGST and pIB1/1 as templates. For the Pex1p(E798Q) variant and for Pex1p(D797N), primer pairs RE4002/RE4003 and RE4686/RE4687 were used, respectively. Cloning of the pRS416-Pex1p(D797Q) construct is described elsewhere^17^. Constructs for expression of Pex6p(E832Q) Walker B mutants were obtained by site-directed mutagenesis using pRSFDuet-HisPex6 and pBM34 as templates with primer pair RE4008/RE4009. All constructs used in this study were confirmed by DNA sequencing.

Plasmid pKE15/2 for expression of GST-Pex15p_1-315_His was constructed as follows. The coding region for cytosolic part of Pex15p (Pex15p_1-315_) was amplified by PCR using primer pair Ku654/Ku1188 with genomic DNA from wild-type *Saccharomyces cerevisiae* strain UTL-7A as template. The PCR-product was digested with *BamHI/XhoI* and cloned into the *BamHI/XhoI* sites of vector pET21d-GST-His6. Sequences of oligonucleotides are available upon request.

### Protein purification

Expression of recombinant Pex1p and Pex6p proteins was performed in *Escherichia coli* Tuner (DE3) cells (Merck, Darmstadt, Germany) at 20 °C in the presence of 0.4 mM IPTG. Time of expression was 20 h for Pex1p and 5 h for Pex6p. Cells were harvested and homogenized by sonication either separately or as a mixture of Pex1p- and Pex6p-expressing strains. The homogenization was performed in AAA-buffer I (50 mM Tris, 300 mM NaCl, 5 mM MgCl_2_, 5 mM ATP-Na_2_/ADP-Na_2_ and 1 mM DTT, pH 7.4) containing 40 mM imidazole and selected protease inhibitors (1 mM PMSF, 8 mM Antipain, 0.3 mM Aprotinin, 1 mM Bestatin, 10 mM Chymostatin, 5 mM Leupeptin, 15 mM Pepstatin). The homogenate was centrifuged at 45,000xg (rotor SS34) for 60 min. The resulting supernatant contained soluble proteins and was loaded to NiNTA-Agarose (5PRIME, Hamburg, Germany) according to manufacturer’s instructions. The column was washed with 20fold column volume of AAA-buffer I supplemented with 40 mM imidazole and bound proteins were eluted in two steps with AAA-buffer II (50 mM Tris, 300 mM NaCl, 5 mM MgCl_2_, 5 mM ATP-Na_2_/ ADP-Na_2 _and 10 mM DTT, pH 7.4) containing 100 mM and 300 mM imidazole, respectively.

For further purification of the HisPex1pGST/HisPex6p-complex, NiNTA eluates containing Pex1p and Pex6p were loaded onto a Glutathione Agarose 4B column (Macherey-Nagel, Düren, Germany). Unbound proteins were removed by washing of the matrix with 6-fold column volume of AAA-buffer II. The column with bound fusion-proteins was subsequently incubated with thrombin overnight at 4 °C and the released Pex1/6p-complex was separated from the agarose matrix by centrifugation.

GST-Pex15p_1-315_His was expressed in BL21 (DE3) cells (Merck, Darmstadt, Germany) from plasmid pKE15/2. Induction was performed for 5 h at 30 °C in the presence of 0.4 mM IPTG. Cells were harvested and homogenized in AAA-buffer I (without nucleotides, with selected protease inhibitors) by sonication. The supernatant of a 45,000×g centrifugation (rotor SS34, 60 min.) was loaded to Glutathione Agarose 4B (Macherey-Nagel, Düren, Germany). The matrix was washed with 20fold column volume AAA-buffer I (without nucleotides) and bound proteins were eluted with AAA-buffer I comprising 50 mM reduced glutathione. For pull-down assays, the glutathione was removed by dialysis and the eluted Pex15p was loaded again onto glutathione agarose. Thrombin cleavage was performed overnight in presence of 5 mM ATP or ADP and where stated 2.5 mM ATPγS was added.

### Size exclusion chromatography

To obtain the hexameric Pex1/6p complex and to analyze the size of Pex1/6p/Pex15p complexes, gel filtration analysis was performed with an ÄKTApurifier system (GE Healthcare, Freiburg, Germany). For small-scale approaches, 50 μl of recombinant proteins were loaded on Superose^TM^6 PC 3.2/30 column (GE Healthcare, Freiburg, Germany) equilibrated with AAA-buffer II and subsequently 50 μl fractions were collected. For preparative gel filtration, 500 μl of recombinant proteins were subjected to a Superose^TM^6 10/300GL column and 500 μl fractions were collected. Calibration of the columns was performed with the HMW-Gel Filtration Calibration Kit (GE Healthcare, Freiburg, Germany) containing thyreoglobulin (669 kDa), ferritin (440 kDa) and aldolase (158 kDa).

### Miscellaneous

Proteins in polyacrylamide gels were visualized by Coomassie staining according to^45^. Native Pex15p complexes and membrane attached Pex1/6p complexes were isolated according to^23^ in the absence or presence of 5 mM ATP or ADP. Immuno-reactive complexes were visualized using Pex1p-, Pex6p-, Pex5p-, Pex18p-, Pex15p-, Pex12p-, Pex13p-, Pex14p-, Pex17p-, or His6-specific primary antibodies and a IRDye 800CW goat anti-rabbit IgG or IRDye 680 goat anti-mouse secondary antibody (LI-COR Bioscience, Bad Homburg, Germany) followed by detection using the “Infrarot Imaging System” (LI-COR Bioscience, Bad Homburg, Germany). ImageJ or LI-COR Odyssey application software 3.0 was used for signal intensity measurements.

## Additional Information

**How to cite this article**: Grimm, I. *et al.* Nucleotide-dependent assembly of the peroxisomal receptor export complex. *Sci. Rep.* **6**, 19838; doi: 10.1038/srep19838 (2016).

## Supplementary Material

Supplementary Information
