# Supplementary material for: Nucleotide-dependent assembly of the peroxisomal receptor export complex
Source: Sci Rep. 2016 Feb 4;6:19838. doi: 10.1038/srep19838 (PMC4740771; doi:10.1038/srep19838)
Supplement: Supplementary Information [file srep19838-s1.pdf]

Nucleotide dependent assembly of the peroxisomal receptor export complex

Immanuel Grimm, Delia Saffian, Wolfgang Girzalsky, and Ralf Erdmann*

Supplementary information:

Supplementary Figure 1

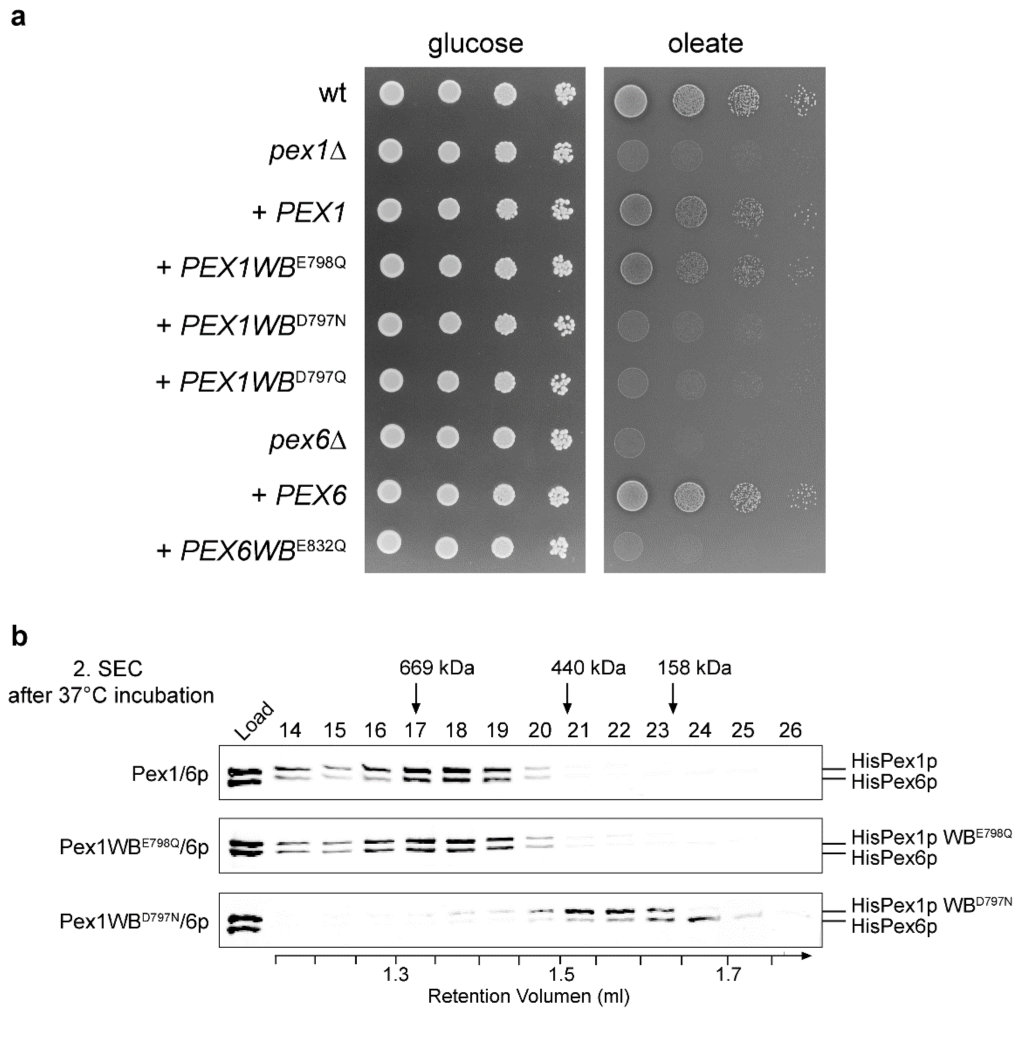

Supplementary Figure 1: Analysis of Walker B mutations of Pex1p and Pex6p for oleate utilization and complex stability

(a) *pex1*Δ and *pex6*Δ yeast strains were functionally complemented with *PEX1* and *PEX6* as well as with Walker B mutations as indicated. A serial dilution of cells was spotted on glucose or oleate containing media and plates were incubated for 2 to 4 days at 30 °C. Deletion of either PEX1 (*pex1*Δ) or PEX6 (*pex6*Δ) and exchange of glutamate to glutamine in Walker B motif of Pex6p (*PEX6WB* ^{E832Q}) resulted in a growth defect on oleate medium. A corresponding mutation in *PEX1* (*PEX1WB* ^{E798Q}) showed no growth defect. Exchange of the adjacent aspartate caused growth defects on oleate (*PEX1WB* ^{D797N}, *PEX1WB* ^{D797Q}).

(b) Recombinant Pex1/6p wild-type, Pex1WB^{D797N}/6p and Pex1WB^{E798Q}/6p complexes were purified from *E.coli* by tandem affinity chromatography and size exclusion chromatography.

Equal amounts of hexameric complexes were incubated at 37 °C for 10 min to trigger ATP hydrolysis and subsequently proteins were subjected to a second size exclusion chromatography. Samples of obtained fractions were analyzed by SDS-PAGE and immunoblotting using αHis-antibodies. Pex1/6p wild-type as well as Pexp1WB^{E798Q}/6p complexes maintain a hexameric arrangement, whereas Pex1WB^{D797N}/6p dissociated into trimeric Pex1p and monomeric Pex6p.
